# Rational combination of SHP2 and mTOR inhibition for the treatment of hepatocellular carcinoma

**DOI:** 10.1002/1878-0261.13377

**Published:** 2023-02-09

**Authors:** Antonio Mulero‐Sánchez, Christel F. A. Ramirez, Aimée du Chatinier, Hui Wang, Sofie J. I. Koomen, Ji‐Ying Song, Marnix H. P. de Groot, Cor Lieftink, Astrid Bosma, Artur Burylo, Olaf van Tellingen, Roderick L. Beijersbergen, Cun Wang, Leila Akkari, René Bernards, Sara Mainardi

**Affiliations:** ^1^ Division of Molecular Carcinogenesis, Oncode Institute The Netherlands Cancer Institute Amsterdam The Netherlands; ^2^ Division of Tumor Biology and Immunology, Oncode Institute The Netherlands Cancer Institute Amsterdam The Netherlands; ^3^ State Key Laboratory of Oncogenes and Related Genes, Shanghai Cancer Institute, Renji Hospital Shanghai Jiao Tong University School of Medicine China; ^4^ Department of Experimental Animal Pathology The Netherlands Cancer Institute Amsterdam The Netherlands; ^5^ Division of Pharmacology The Netherlands Cancer Institute Amsterdam The Netherlands

**Keywords:** combination, hepatocellular carcinoma, mTOR, receptor tyrosine kinases, SHP2, therapy

## Abstract

Liver cancer is the fourth most common cause of cancer‐related death worldwide, with hepatocellular carcinoma (HCC) being the main primary malignancy affecting the liver. Unfortunately, there are still limited therapeutic options for HCC, and even the latest advances have only increased the overall survival modestly. Thus, new treatment strategies and rational drug combinations are urgently needed. Reactivation of receptor tyrosine kinases (RTK) has been described as a mechanism of intrinsic resistance to targeted therapies in a variety of cancers, including inhibitors of mTOR. The design of rational combination therapies to overcome this type of resistance is complicated by the notion that multiple RTK can be upregulated during the acquisition of resistance. SHP2, encoded by the gene *PTPN11*, acts downstream of virtually all RTK, and has proven to be a good target for small molecule inhibitors. Here, we report activation of multiple RTK upon mTOR inhibition in HCC which, through SHP2, leads to reactivation of the mTOR pathway. We show that co‐inhibition of both mTOR and SHP2 is highly synergistic *in vitro* by triggering apoptosis*.* More importantly, the combination is well‐tolerated and outperforms the monotherapies in impairing tumor growth in multiple HCC mouse models. Our findings suggest a novel rational combination therapy for the treatment of HCC.

## Graphical abstract

We show that, upon mTOR inhibition with AZD8055, there is upregulation of several receptor tyrosine kinases. As consequence, SHP2 signals through the mTOR pathway. Hence, dual blockade of SHP2 and mTOR can effectively suppress the mTOR pathway and drive anti‐cancer response, both *in vitro* and *in vivo*, in several hepatocellular carcinoma models.

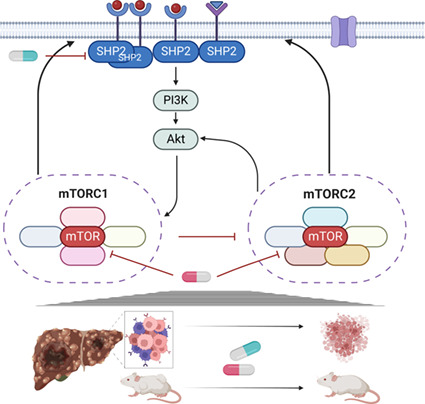

**Abbreviations**
BRAF: v‐raf murine sarcoma viral oncogene homolog B1
CRISPR: clustered regularly interspaced short palindromic repeats
ERK: extracellular signal‐regulated kinase
FDA: U.S. Food and Drug Administration
HCC: hepatocellular carcinoma
H&E: hematoxylin and eosin
KRAS: Kirsten rat sarcoma viral oncogene homolog
MEK: mitogen‐activated protein kinase kinase
mTOR: mammalian/mechanistic target of rapamycin
NSCLC: non‐small‐cell lung cancer
PROTAC: proteolysis targeting chimera
PTPN11: protein tyrosine phosphatase non‐receptor type 11
qRT‐PCR: quantitative real‐time polymerase chain reaction
RTK: receptor tyrosine kinases
SHP2: Src homology 2 (SH2) domain‐containing tyrosine phosphatase‐2
TNCB: triple negative breast cancer

## 1 Introduction

Hepatocellular carcinoma (HCC) is the most common primary liver tumor [1, 2, 3]. The incidence of this cancer is particularly high in Asia [4] and is rising in western countries, mainly due to an increase in Hepatitis C and alcohol abuse [5], making HCC the fourth most common cancer‐related cause of death worldwide [6]. This is explained, at least in part, by the lack of effective treatment options, since only a small number of patients are eligible for surgery and systemic chemotherapy has poor efficacy [7]. Currently approved therapies for HCC include several multi‐kinase inhibitors, such as sorafenib, regorafenib, lenvatinib and cabozantinib, some of which are used clinically in combination with checkpoint immunotherapy [8].

The mTOR pathway is the second most altered signaling cascade in cancer [9]. Several clinical trials have studied the efficacy of mTOR inhibitors in different tumors, including breast cancer [10] and HCC (NCT01079767, NCT00467194). The results of these studies have been disappointing due to emerging mechanisms of resistance to the therapy [11]. Several small molecules have been developed aiming to improve on the first‐generation rapamycin‐based mTOR inhibitors. This resulted into drugs with high specificity that inhibit both the mTORC1 and mTORC2 complexes [12]. The dual mTOR1/2 kinase inhibitor, AZD8055, is a highly potent ATP‐competitive inhibitor that impairs the activity of mTORC1/2 complexes as well as mTOR‐mediated AKT activity [2, 13]. Unfortunately, *in vivo* models of breast cancer treated with this or similar inhibitors have identified the induction of acquired resistance, mainly due to activation of HER2 receptor tyrosine kinase. Consequently, the combination of the HER2 inhibitor lapatinib with the mTOR inhibitor AZD8055, impairs breast cancer tumor growth *in vivo* [14].

Resistance to targeted therapies via activation of receptor tyrosine kinases is not uncommon [15]. In the last decade, we have learned this is a recurrent mechanism of acquired resistance to agents such as MEK, BRAF and mTOR inhibitors in several solid tumors [14, 16, 17]. Mechanistically, the upregulation of receptor tyrosine kinases (RTK) can recruit and hyperactivate SHP2, a tyrosine phosphatase encoded by the *PTPN11* gene, which in turn activates downstream effectors. This leads to a positive feedback‐loop within the targeted pathway [18]. Our group and others have shown that SHP2 blockade in combination with MEK inhibitors could be beneficial for patients harboring *KRAS* mutant pancreatic [19], non‐small‐cell lung cancer (NSCLC) [20], serous ovarian and triple negative breast cancer (TNBC) [21] tumors as well as *KRAS* WT‐amplified gastroesophageal tumors [22]. In liver cancer, SHP2 appears to have a dual role: it acts as a tumor suppressor during initiation [23] but its overexpression is a biomarker for progression and poor prognosis in advanced liver cancer patients [24]. More recently, while exploring the use of mTOR inhibitors as senolytic agents in senescent HCC cells [25], we reported that SHP2 is activated in proliferating HCC cells treated with AZD8055.

We show here that a combination of mTOR and SHP2 inhibitors is highly synergistic in multiple human and mouse HCC models *in vitro* and observed a synergistic induction of apoptosis. Moreover, we demonstrate that the combination is capable of controlling tumor growth and increasing survival in an aggressive immunocompetent *in vivo* model of liver cancer, in the absence of toxicity. These results provide a strong preclinical rationale for further clinical exploration of combined mTOR and SHP2 inhibition for the treatment of hepatocellular carcinoma.

## 2 Materials and methods

### 2.1 Cell lines

The human liver cancer cell lines Hep3B, Huh7 and SNU398 were provided by Erasmus University (Rotterdam, Netherlands). MHCC97H was provided by the Liver Cancer Institute of Zhongshan Hospital (Shanghai, China). The liver cancer cell lines were established from hepatocellular carcinoma subtype of liver cancer (HCC). HCC cancer cells were cultured in DMEM with 10% FCS, glutamine and penicillin/streptomycin (Gibco®) at 37 °C/5% CO_2_. The liver cancer cell lines were authenticated by applying short tandem‐repeat DNA profiling. A mouse liver cancer cell line with specific genetic background (*Myc* ^OE^;*Trp53* ^−/−^) was generated by the Akkari Lab (Christel F. A. Ramirez) (Netherlands Cancer Institute) and was extracted and grown in collagen‐coated plates as described previously [26]. Mycoplasma contamination was excluded via a PCR‐based method.

### 2.2 Compounds and antibodies

AZD8055 (S1555), RMC‐4550 and SHP099 were purchased from Selleck Chemicals (Houston, TX, USA). Compound #57 is covered by a patent application (WO 2015/107495A1) and was synthesized as described previously [27]. Primary antibodies against HSP90 (sc‐7947, sc‐13 119) and SHP2 (sc‐280) and c‐MYC (sc‐764) were purchased from Santa Cruz Biotechnology (Dallas, TX, USA). Antibody against p‐SHP2 (ab62322) was obtained from Abcam (Cambridge, UK). Antibodies against GAPDH (#5174), SHP2 (#3752), p‐SHP2 (#3751), p‐S6RP (#2211, #5364), S6RP (#2217), p‐4EBP1 (#9456, #2855) and 4EBP1 (#9644) were purchased from Cell Signaling Technology (Leiden, The Netherlands). Antibodies against alpha‐Tubulin (T9026) and vinculin (V9131) were purchased from Sigma‐Aldrich (Darmstadt, Germany). Primary antibody against p‐c‐MYC (S62) was purchased from AbCam (ab51156).

### 2.3 RNA sequencing

Cells were plated in 10‐cm plates at a density of 1 000 000–2 000 000 cells, depending on growth rate. Cells were treated with the drugs of interest for 24 h (MHCC97H) or 48 h (Hep3B and Huh7). The library was prepared using the TruSeq RNA Sample Preparation kit (Illumina, Eindhoven, The Netherlands). Sequences were aligned against the human genome (hg38) and enrichment analysis was performed using gene set enrichment analysis (gsea) software [28, 29]. A custom gene set of 58 RTK was used to assess the enrichment of RTK expression in the presence of mTOR inhibition. The PENG_RAPAMYCIN_RESPONSE_DN gene set was used to evaluate the enrichment mTOR signaling in the presence of mTOR inhibitors. The 50 hallmark gene set from GSEA was used to assess transcriptional activation of cancer‐related pathways in an unbiased manner [30]. The GSEA was done using the r‐package fgsea version 1.18.0. (Bioconductor, Buffalo, NY, USA) NES score is depicted by color coding. The package calculates an ES‐score and a *P*‐value based on an empirical NULL distribution [31].

### 2.4 Human phospho‐receptor tyrosine kinase array

Cells were plated in 10‐cm plates at a density of 1 000 000–2 000 000 cells depending on growth rate, and stimulated with the drugs of interest for 48 h. At the indicated time points, cells were washed with ice‐cold PBS, and Phospho‐RTK analysis was performed using the Human Phospho‐Receptor Tyrosine Kinase Array Kit (R&D Systems, Minneapolis, MN, USA).

### 2.5 Protein lysate preparation and western blots

Cells were plated in 10‐cm plates at a density of 250 000–1 000 000 cells, depending on growth rate. After 24 h, cells were grown in serum‐free DMEM to synchronize the cell cycle. After overnight starvation cells were stimulated with 10% FCS and the drugs of interest. At the indicated time points, cells were washed with ice‐cold PBS and lysed in radioimmunoprecipitation assay buffer supplemented with 1× Halt™ Protease and Phosphatase Inhibitor Cocktail (100x) (ThermoFisher Scientific, Breda, The Netherlands). Normalization of protein concentration was performed using the Pierce™ BCA Protein Assay Kit (ThermoFisher Scientific). Normalized protein samples were reduced using 1× Bolt™ Sample Reducing Agent (ThermoFisher Scientific, 10×) and 1× Bolt™ LDS Sample Buffer (ThermoFisher Scientific, 4×). Loading samples were resolved by electrophoresis in Bolt 4–12% Bis‐Tris Plus Gels (ThermoFisher Scientific) submerged in MOPS running buffer followed by Western blotting. Antibodies against vinculin, alpha‐Tubulin, p‐SHP2, p‐4EBP1, and 4EBP1, HSP90, SHP2, p‐S6RP and S6RP were diluted in 1 : 1000. Antibody against GAPDH was diluted in 1 : 5000. HRP‐conjugated secondary antibodies were diluted 1 : 10 000.

### 2.6 Colony formation assays

Cells were plated into 96‐well plates at a density of 1000–4000 cells per well, depending on growth rate. Drugs were added at the indicated concentrations 24 h later using the HP D300 Digital Dispenser (HP). Phenylarsine oxide and DMSO were used as a positive and negative control, respectively. After 5–7 days, cells were fixed with 4% paraformaldehyde in PBS and stained with 0.1% crystal violet in water.

### 2.7 CellTiter‐Blue® cell viability assay

Cells were plated into 96‐well plates at a density of 3000–6000 cells per well, depending on growth rate. Drugs were added at the indicated concentrations 24 h later using the HP D300 Digital Dispenser (HP). PAO and DMSO were used as a positive and negative control, respectively. After 72 h, 5× CellTiter‐Blue® Reagent (20×) was added directly to culture medium and plates were incubated at 37 °C for 4 h to allow metabolic reduction of resazurin (dark blue) to resorufin (red, fluorescent) by viable cells. Fluorescence was measured at 560_Ex_/590_Em_. Percentage of viability was calculated by subtracting the mean viability score of the positive control (PAO) from the mean cell viability score of either treatment regimen divided by the mean viability of the negative control (DMSO).

### 2.8 IncuCyte® cell proliferation and apoptosis assay

Cells were plated into 96‐well plates at a density of 1000–4000 cells per well, depending on growth rate. Drugs were added to the culture medium at the indicated concentrations 24 h later using the HP D300 Digital Dispenser (HP). PAO and DMSO were used as a positive and negative control, respectively. For quantification of cell apoptosis, 5 μm IncuCyte® Caspase‐3/7 Green Apoptosis Assay Reagent (Essen Bioscience) was added. This reagent couples a caspase 3/7 recognition motif (DEVD) to NucView™488, a DNA intercalating dye that fluorescently labels the nuclear DNA once cleaved in cells undergoing caspase 3/7‐mediated apoptosis. Cells were imaged every 4 h for a total of 72 h in IncuCyte ZOOM (Essen Bioscience, Newark, UK), and phase‐contrast images were analyzed to detect cell proliferation based on cell confluence. Apoptosis was quantified by dividing the Phase Object Confluence value (percent) by the Green Object Confluence value (percent) provided by the incucyte zoom software.

### 2.9 Drug combination synergy scoring (BLISS score)

Indicated cell lines were seeded into 96‐well plates at a density of 1750–5000 cells per well, depending on growth rate. After a 24 h cells were grown in the absence of FCS (starvation) overnight. After starvation, a 9 × 6 matrix of drug concentrations was added in a row‐wise and column‐wise 1 : 2 dilution series, using the HP D300 Digital Dispenser (HP). Cells were cultured with the drugs for 6–7 days at 37 °C and medium containing the indicated drugs. Medium was refreshed after 72 h. CellTiter‐Blue was added to the plates and after 2–4 h of incubation, cell viability was measured according to manufacturer's instructions with the EnVision multimode plate reader. Treatment with 10 μm PAO and 0.2% DMSO was used as a positive and a negative control, respectively. Cells were fixed with 4% PFA in PBS and stained with 0.5% crystal violet in water. After staining, crystal violet was dissolved in 30% acetic acid for 15 min and measured at 544 nm using an EnVision multimode plate reader. The synergy experiments were performed in triplicate. For each replicate and concentration combination the expected additive effect value was calculated based on the BLISS independence model [32]. This value was subtracted from the measured value, resulting in a delta value. This delta value was also calculated as a percentage of the expected value. Finally, a one‐sided *t*‐test was performed to test whether the measured values were significantly higher than the expected additive values. Highlighted with a green background are the combinatorial concentrations with a *P*‐value ≤ 0.05 and a delta value ≥ 10%. The delta value, percentage and *P*‐value are depicted in the figures.

### 2.10 qRT‐PCR

Cells were plated into 6‐well plates at a density of 50 000–200 000 cells per well, depending on growth rate, and stimulated with the indicated drugs 24 h later. At the indicated time points, cells were washed with ice‐cold PBS, and total RNA was extracted using the ISOLATE II RNA Mini Kit (Bioline). Normalization of RNA concentration was performed, and cDNA was synthesized using the SensiFAST cDNA Synthesis Kit (Bioline, Waddinxveen, The Netherlands). qPCR reactions were performed with the SensiFAST SYBR Lo‐ROX Kit (Bioline). Relative expression was determined by calculating the delta delta ct value with GAPDH as housekeeping gene. The sequences of the primers used for qRT‐PCR analyses are described in Table 1.

**Table 1:** Primers.

Primer	Sequence
AXL_Forward	GTGGGCAACCCAGGGAATATC
AXL_Reverse	GTACTGTCCCGTGTCGGAAAG
ERBB3_Foward	GACCCAGGTCTACGATGGGAA
ERRB3_Reverse	GTGAGCTGAGTCAAGCGGAG
DDR1_Forward	AAGGGACATTTTGATCCTGCC
DDR1_Reverse	CCTTGGGAAACACCGACCC
EPHA2_Forward	TGGCTCACACACCCGTATG
EPHA2_Reverse	GTCGCCAGACATCACGTTG
EPHB3_Forward	TGGGTAACATCTGAGTTGGCG
EPHB3_Reverse	TGGTATGTGCGGATGGGATTC
ERBB2_Forward	TGCAGGGAAACCTGGAACTC
ERBB2_Reverse	ACAGGGGTGGTATTGTTCAGC
FGFR3_Forward	TGCGTCGTGGAGAACAAGTTT
FGFR3_Reverse	GCACGGTAACGTAGGGTGTG
INSR_Forward	AAAACGAGGCCCGAAGATTTC
INSR_Reverse	GAGCCCATAGACCCGGAAG
IGF1R_Forward	TCGACATCCGCAACGACTATC
IGF1R_Reverse	CCAGGGCGTAGTTGTAGAAGAG
PDGFRA_Forward	TGGCAGTACCCCATGTCTGAA
PDGFRA_Reverse	CCAAGACCGTCACAAAAAGGC
PDGFRB_Forward	AGCACCTTCGTTCTGACCTG
PDGFRB_Reverse	TATTCTCCCGTGTCTAGCCCA
GAPDH_Forward	GGAGCGAGATCCCTCCAAAAT
GAPDH_Reverse	GGCTGTTGTCATACTTCTCATGG

### 2.11 CRISPR–Cas9‐mediated gene knockout

CRISPR–Cas9‐based *PTPN11*‐knockout cell populations were obtained as previously described [18]. First, we introduced a vector expressing inducible Cas9 and after selection with blasticidine we introduced PTPN11 gRNA_B vector. We then selected with puromycin for cells with the gRNA_B and induced Cas9 expression with doxycycline. Expression of SHP2 total levels was measured by western blot.

### 2.12 Statistics

All *in vitro* data are expressed as averages (mean ± SD) from at least three technical replicates, and they have been independently reproduced at least twice with similar results (except for RNA sequencing and the Human Phospho‐Receptor Tyrosine Kinase Array). Statistical significance (*P*‐value) was determined as indicated in the figure legends. *P*‐values below 0.05 were considered statistically significant.

### 2.13 Xenografts

All animals were manipulated according to protocols approved by the Shanghai Medical Experimental Animal Care Commission and Shanghai Cancer Institute. MHCC97H cells (5 × 10^6^ cells per mouse) were injected subcutaneously into the right posterior flanks of 6‐week‐old BALB/c nude mice (male, 6–10 mice per group). Tumor volume based on caliper measurements was calculated by the modified ellipsoidal formula: tumor volume = ½ length × width. After tumor establishment, mice were randomly assigned to 6 days·week^−1^ treatment with vehicle, AZD8055 (10 mg·kg^−1^), oral gavage, dissolved in 5% DMSO +30% PEG300 + ddH2O), SHP099 (37.5 mg·kg^−1^, oral gavage, dissolved in 0.5% (w/v) hydroxypropyl methylcellulose in water, or a drug combination in which each compound was administered at the same dose and schedule as a single agent. Mice were obtained from Vital River Laboratory Animal Technology Co., Ltd. All animals were manipulated according to protocols approved by the Shanghai Medical Experimental Animal Care Commission and Shanghai Cancer Institute. The environmental enrichment was established as previously described [33]. Animals were housed in micro‐isolator cages of dimensions 30.5 × 19 × 14 cm, including a wire rack in the cage for holding food and a water bottle. Animals were housed on a 12‐h light/dark cycle. Animal license number: SYXK‐2017‐0011.

### 2.14 Immunocompetent HCC murine models

*Myc* ^*OE*^ *Trp53* ^*KO*^ HCC were generated by hydrodynamically injecting 0.9% sterile NaCl solution/plasmid mix containing 5 μg pT3‐MYC (Addgene 92046, Teddington, UK), 5 μg pX330‐p53 (Addgene 59910) and 2.5 μg CMV‐SB13 transposase at a final volume of 10% of the bodyweight in the lateral tail vein of 6‐ to 8‐week‐old C57BL/6 female mice [26] (Janvier Labs). Two weeks after hydrodynamic tail vein injection (HDTVi), mice were monitored by weekly MRI, as previously described [26]. When HCC tumor nodules were visible by MRI (14–21 days post HDTVi), tumor‐size‐matched mice were randomized across treatment groups. Mice received daily doses of vehicle, AZD8055 (10 mg·kg^−1^), RMC‐4550 (30 mg·kg^−1^) or a combination of both drugs via oral gavage until endpoint symptoms emerged (tumor volume ≤ 2 cm^3^). All animal studies were reviewed and approved by the NKI Animal Welfare Body. Mice used in this experiment are from Janvier Labs, France. The 6‐ to 8‐week‐old female C57BL/6J mice were kept in individually ventilated cages. Food and water were provided *ad libitum*. All animal procedures were reviewed and approved by the Animal Ethics Committee of the Netherlands Cancer Institute. Animal license number: 24.2.10207.

### 2.15 Histopathology

Immunocompetent somatic (*Myc* ^*OE*^ *Trp53* ^*KO*^) HCC‐bearing mice were perfused with PBS. Livers containing tumors were dissected and fixed in formalin and processed as described previously [26]. Paraffin‐embedded tumor samples were sectioned at a thickness of 2 μm and stained with hematoxylin and eosin (H&E) according to standard procedures. The necrotic (including hemorrhagic) areas were estimated as a percentage of total tumor volume presented in the H&E sections.

### 2.16 Flow cytometry

Once endpoint was reached (tumor volume ≥ 2000 mm^3^), *Myc* ^*OE*^ *Trp53* ^*KO*^ tumor‐bearing mice were intracardially perfused with 10 mL PBS and livers bearing HCC nodules were macro‐dissected. Single‐cell suspensions of individual HCC nodules were prepared using the Liver Dissociation kit (Miltenyi Biotec) and the gentleMACS Octo Dissociator, following the manufacturer's instructions. Cell suspensions were stained with the indicated antibodies (Table S1) and fixed with eBioscience fixation and permeabilization kit (Invitrogen) as previously described [26]. A five‐laser Aurora spectral flow cytometer (Cytek Biosciences) was used for flow cytometry analyses to determine the proportions of myeloid and lymphoid populations using flowjo® software as previously described [26]. All antibodies for flow cytometry were titrated in a lot‐dependent manner and are listed in Table S1.

## 3 Results and Discussion

### 3.1 Feedback reactivation of receptor tyrosine kinases upon mTOR inhibition in HCC

**Fig. 1:**
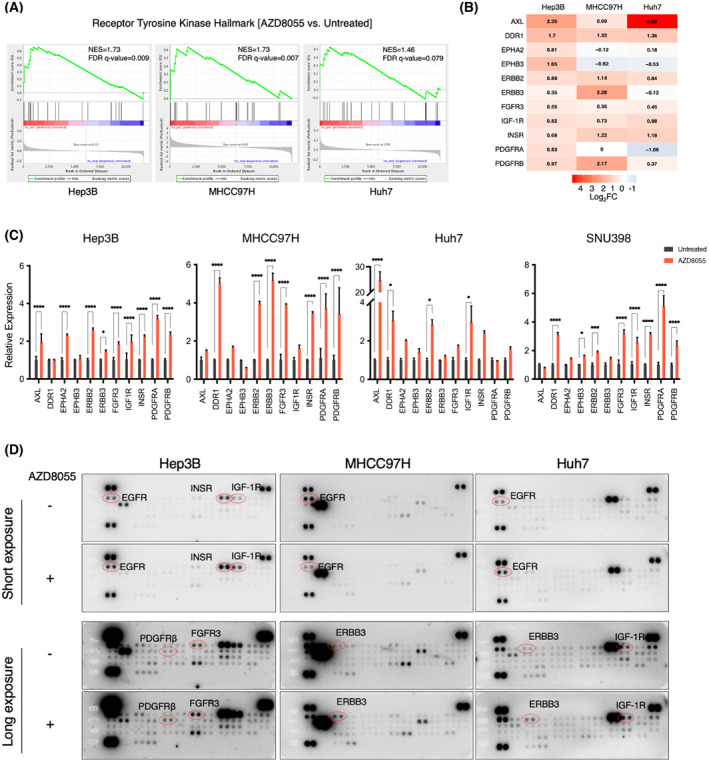
Feedback reactivation of receptor tyrosine kinases upon mTOR inhibition in HCC. (A) Hep3B, Huh7 and MHCC97H were treated with 100 nm AZD8055 for either 24 h (MHCC97H) or 48 h (Hep3B and Huh7), RNA was extracted, sequenced and Gene set enrichment analyses (GSEA) were performed. A custom gene set of 58 RTK was used to assess the enrichment of RTK expression. Enrichment scores (ES), normalized enrichment scores (NES) and *P*‐values are reported (*n* = 1). GSEA from Subramanian determined the *P*‐value for an ES score using a NULL distribution of ES scores from rounds of randomizing the data. (B) Heatmap highlighting abundance of mRNA levels of individual genes in Hep3B, Huh7 and MHCC97H cells treated with AZD8055 vs. untreated cells (log2‐fold change) (*n* = 1). (C) Hep3B, Huh7, MHCC97H and SNU398 cells were treated with AZD8055 (100 nm) for 48 h prior to mRNA extraction, and qRT‐PCR analysis of selected genes was performed. Mean value of a representative experiment performed in technical triplicate; the error bars represent standard deviation. Statistics using a two‐way ANOVA test. *P < 0.05; **P < 0.01; ***P < 0.001; ****P < 0.0001 (*n* = 2). (D) Hep3B, Huh7 and MHCC97H cells were treated with AZD8055 (100 nm) for 48 h, and extracted proteins were analyzed using a Human Phospho‐Receptor Tyrosine Kinase Array Kit. Tyrosine‐protein kinase receptor UFO (AXL), discoidin domain receptor family, member 1 (DDR1), ephrin type‐A receptor 2 (EPHA2), ephrin type‐B receptor 3 (EPHB3), human epidermal growth factor receptor 2 (ERBB2/HER2), human epidermal growth factor receptor 3 (ERBB3/HER3), fibroblast growth factor receptor 3 (FGFR3), insulin‐like growth factor 1 receptor (IGF1R), insulin receptor (INSR), platelet‐derived growth factor receptor A (PDGFRα), platelet‐derived growth factor receptor B (PDGFRβ), epidermal growth factor receptor (EGFR) (*n* = 2).

Similar to what has been described previously in breast tumors [14], we observed an increased expression and activation of multiple receptor tyrosine kinases upon mTOR inhibition in HCC cell lines. RNA sequencing analysis of Hep3B, MHCC97H and Huh7 cells, either untreated or treated with 100 nm of AZD8055 for 24 h (MHCC97H) or 48 h (Hep3B and Huh7), revealed an overall increased transcription of RTK, as judged by a custom gene set of 58 RTK [28, 29] (Fig. 1A). Of note, we observed that the kinetics of feedback reactivation were different, depending on the cellular model, occurring faster in MHCC97H cells. Eleven tyrosine kinase receptors showed increased transcription in at least one of the three cell lines: AXL, DDR1, EPHA2, EPHB3, ERBB2, ERBB3, FGFR3, IGF‐1R, INSR, PDGFRA and PDGFRB (Fig. 1B). This observation was validated by qRT‐PCR, which included a fourth cell line: SNU398 (Fig. 1C). These data show that mTOR inhibition induces an increased transcription of multiple receptor tyrosine kinases in HCC cell lines, with different cell lines having individual preferences in terms of which RTK is upregulated. Next, we performed phospho‐RTK immunoblots to check protein activation after 48 h of mTOR inhibition. The mTOR inhibition induced increased phosphorylation of multiple RTK, correlating overall with the transcriptomic data (Fig. 1D). Again, heterogeneous activation of RTK was observed between different cell lines. For example, EGFR showed increased phosphorylation in Hep3B and in Huh7, whereas this was decreased in MHCC97H. On the other hand, multiple RTK were upregulated within the same cell model, suggesting the possibility of autocrine activation of RTK [34, 35]. To explore the functional consequences of AZD8055‐induced RTK upregulation, we performed unbiased GSEA analyses of 50 gene sets representing hallmarks of cancer [30]. We observed that, upon treatment with AZD8055, KRAS signaling was significantly upregulated, whereas mTOR signaling and MYC targets were significantly decreased (Fig. S1A).

Overall, these data indicate that, shortly following mTOR inhibition, the mTOR pathway is downregulated, but feedback upregulation of RTK was observed. This in turn activates KRAS, which can potentially initiate downstream signaling cascades. Unfortunately, the heterogeneity in activation of RTK hinders the co‐inhibition of specific RTK together with AZD8055, preventing feedback reactivation of downstream pathways.

### 3.2 SHP2 mediates the reactivation of the mTOR pathway upon inhibition with AZD8055

**Fig. 2:**
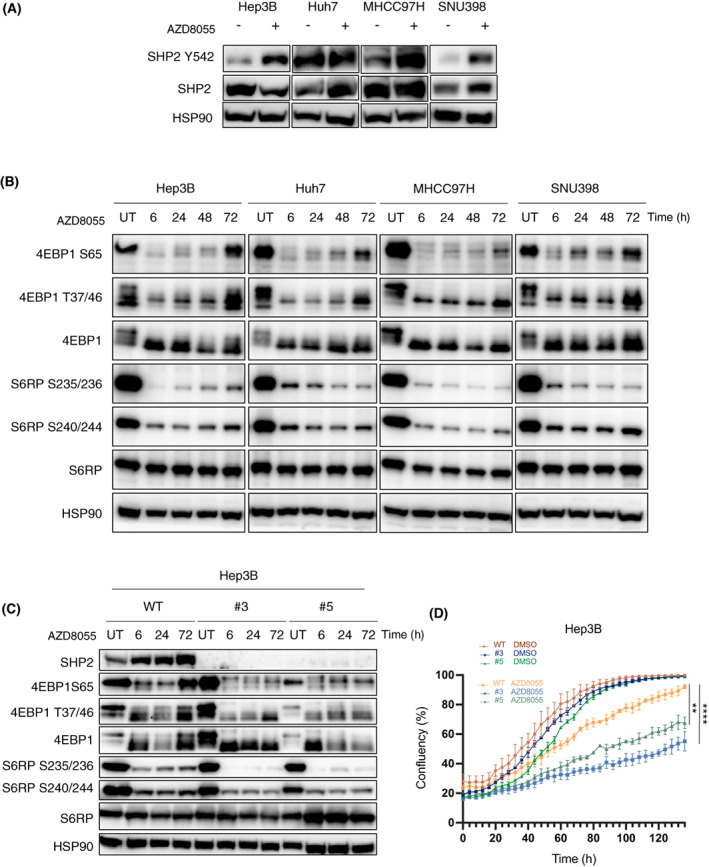
SHP2 plays a role in the reactivation of the mTOR pathway upon mTOR inhibition with AZD8055. (A) Hep3B, Huh7, MHCC97H and SNU398 cells were treated with AZD8055 (50–100 nm), and cell lysates including those from untreated (UT) cells were collected at 48 h to blot for the indicated antibodies (*n* = 3). (B) Western blot analyses for the indicated antibodies. Hep3B, Huh7, MHCC97H and SNU398 cells were treated with AZD8055 (100 nm) and cell lysates were collected at the indicated time points. Cell lysates from untreated cells were collected at 24 h. HSP90 was used as loading control (*n* = 2). (C) Hep3B WT and *PTPN11 knock‐out* (KO) cells were treated with 100 nm AZD8055. Samples were collected at indicated time points to blot for the indicated antibodies. Untreated samples were collected at 24 h (*n* = 2). (D) Growth curves for Hep3B WT and Hep3B *PTPN11 KO* cells in DMSO or in the presence of AZD8055. Phase‐contrast images were taken every 4 h and cell proliferation was determined based on cell confluency. Graphs show mean ± standard deviation, *n* = 3. Statistical analyses using unpaired *t*‐test. **P < 0.01; ****P < 0.0001.

Hyperactivation of RTK as a consequence of downstream pathway inhibition can lead to the reactivation of the inhibited pathway, enabling tumor cells to become resistant to the therapy. This has been reported previously in NSCLC and PDAC [14, 19, 20, 36] tumors upon MEK or ERK inhibition or in the context of breast tumors upon mTOR inhibition. Since SHP2 has been shown to mediate signal transduction downstream of most RTK [37, 38, 39, 40, 41], we checked whether SHP2 activity was increased upon 48 h treatment with AZD8055 in HCC cell lines. Three of four cell lines tested, increased phosphorylation of Y542 of SHP2 (Fig. 2A), consistent with the activation of RTK observed previously (Fig. 1). We then asked whether the RTK upregulation could ultimately lead to mTOR pathway reactivation over time. For this, we performed western blot analysis at different time points (6–72 h) after treatment with AZD8055 and looked for phosphorylation levels of two classical mTOR effectors, 4EBP1 and S6RP. Of note, whereas 4EBP1 has been mainly described as an mTOR target, S6RP can also be phosphorylated by the MAPK downstream effector RSK1 [42]. As shown in Fig. 2B, 4EBP1 phosphorylation was increased at 72 h in all the HCC cell models tested. On the other hand, S6RP phosphorylation levels were not affected in some cell lines, suggesting that the feedback reactivation affects mTOR more than MAPK downstream signaling does. To check whether SHP2 activity is essential for the observed reactivation of the mTOR pathway, we generated *PTPN11* knockouts in Hep3B (clones #3 and #5) and MHCC97H (clones and #2 and #6) cells (Figs 2C,D and S2). First, we evaluated mTOR activity over time in WT cells and *PTPN11* KO cells after AZD8055 treatment (Figs 2C and S2A). Semiquantitative measures of 4EBP1 and S6RP phosphorylation sites showed an overall decrease of mTOR activity in the SHP2 KO populations in the presence of the mTOR inhibitor (Fig. S2C), suggesting that SHP2 mediates reactivation of the mTOR pathway upon AZD8055 treatment. Of note, SHP2 facilitates the transmission of signal from the RTK to multiple oncogenic pathways, such as the RAS‐MAPK and PI3K‐AKT–mTOR pathways [21, 38, 43, 44]. Here, we decided to focus on the reactivation of the same mTOR pathway, as modulation of the common MAPK and mTOR target S6RP was not as prevalent as the mTOR exclusive target 4EBP1.

As a second validation step, we performed quantitative proliferation assays and showed that the SHP2 KO cells were more sensitive to AZD8055 than were the WT cells (Fig. 2D and Fig. S2B). These data suggest that SHP2 impairment can prevent feedback reactivation of the mTOR pathway, thus leading to increased sensitivity to mTOR inhibition.

### 3.3 The combination of mTOR and SHP2 inhibitors is synergistic and triggers apoptosis *in vitro*

**Fig. 3:**
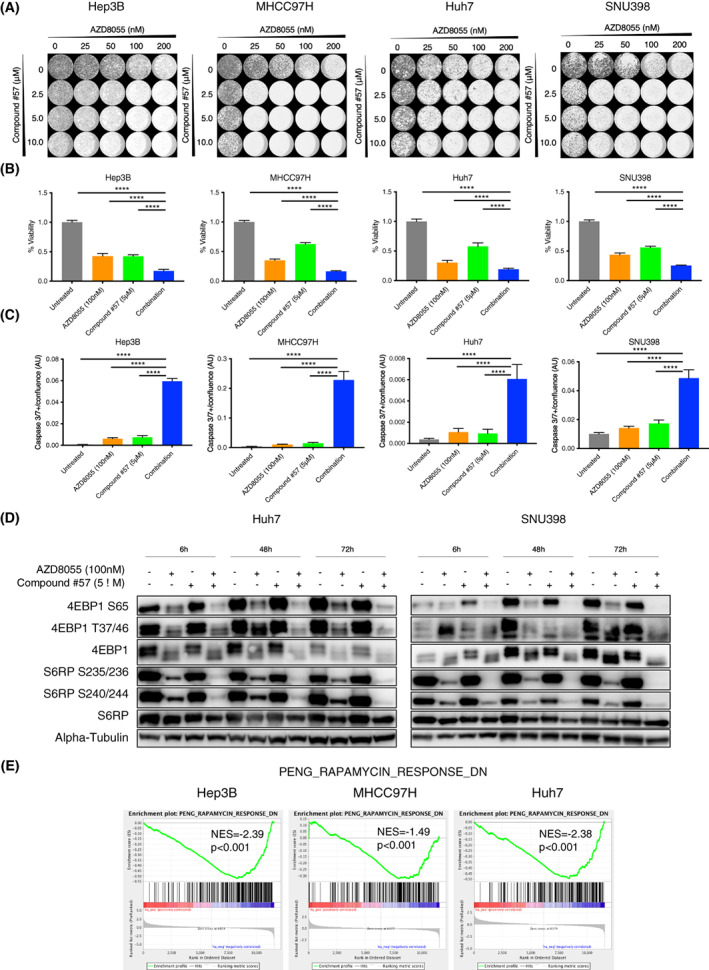
Combination of mTOR and SHP2 inhibitors is synergistic and triggers apoptosis *in vitro*. (A) Long term proliferation assay (5–7 days) of Hep3B, Huh7, MHCC97H, and SNU398 cells treated with increasing doses of AZD8055, an SHP2 inhibitor (compound #57) or a combination of both (*n* = 3). (B) Cell viability assay (72 h), in the indicated cell lines, for: DMSO, monotherapies and combination at the indicated doses. Error bars indicate standard deviation (SD). Statistical analyses using a one‐way ANOVA test, ****P < 0.0001 (*n* = 3). (C) Caspase 3/7‐mediated apoptosis analyses for cells treated at the indicated doses for 72 h. Combination = AZD8055 (100 nm) plus Compound #57 (5 μm). Error bars indicate standard deviation (SD). Statistical analyses using a one‐way ANOVA test, ****P < 0.0001 (*n* = 3). (D) Western blot analyses for the indicated antibodies. Comparison of non‐treated cells or cell streated with the indicated drugs at the indicated time points (*n* = 3). (E) Hep3B and Huh7 cells were treated with AZD8055 (100 nm) alone or in combination with compound #57 (5 μm) for 48 h, and RNA sequencing was performed followed by GSEA. MHCC97H cells were treated for 24 h prior to sequencing. The PENG_RAPAMYCIN_RESPONSE_DN gene set [49] was used to assess the enrichment of mTOR signaling in the presence of mTOR and SHP2 inhibitors compared with mTOR inhibition alone. Enrichment scores (ES), normalized enrichment scores (NES) and *P*‐values are reported (*n* = 1). GSEA from Subramanian determined the *P*‐value for an ES score using a NULL distribution of ES scores from rounds of randomizing the data.

Multiple approaches have been pursued to block SHP2 activity, including allosteric SHP2 inhibitors [27, 45], PROTAC molecules [46, 47] and protein–protein interaction inhibitors [48]. We first tested compound #57, a previously described SHP2 allosteric inhibitor, suitable for *in vitro* studies [20], by combining multiple doses with increasing concentrations of AZD8055 in a synergy matrix. Colony formation was impaired by the combined treatment at concentrations that were ineffective as monotherapies in multiple cell lines (Fig. 3A). Similarly, a significant decrease in cell viability occurred in the combination treatments compared with monotherapies (Fig. 3B). Moreover, BLISS synergy score analyses performed in two different models using both compound #57 and an additional SHP2 inhibitor, RMC‐4550 [45], confirmed that the combination of SHP2 and mTOR inhibitors is synergistic in HCC (Fig. S3). Notably, both SHP2 inhibitors performed very similarly in the BLISS score analyses, suggesting both drugs are acting on‐target. To distinguish between a possible cytotoxic or cytostatic effect of the combination, we performed a Caspase 3/7 assay to monitor the induction of apoptosis over 72 h (Figs 3C and S4). The results show that the combination potently triggers apoptosis in all models tested, in a pattern that is inversely correlated with the cell viability quantified in Fig. 3B. Notably, although the monotherapies significantly decreased cell viability, albeit to a lesser extent than the combination, they did not trigger apoptosis, suggesting that whereas inhibition of SHP2 or mTOR alone only has a modest cytostatic effect, the combination can trigger an apoptotic response, effectively eliminating cancer cells. Next, we checked the effect of SHP2 and mTOR inhibitors, either alone or in combination, at the biochemical level. Interestingly, sustained mTOR pathway inhibition was observed with the dual treatment of AZD8055 plus compound #57, as judged by phosphorylation of 4EBP1 and S6RP (Fig. 3D). In contrast, AZD8055 monotherapy was not sufficient to suppress mTOR activity at late time points. Finally, we compared the transcriptional signature of mTOR pathway activity in samples treated with the combination vs. samples treated with AZD8055 only. Interestingly, all models exhibited a significant downregulation of the PENG_RAPAMYCIN_DN gene expression signature in the combination as compared with the monotherapies [49]. Additionally, we performed GSEA analyses of 50 gene sets representing hallmarks of cancer [30]. Importantly, when comparing the combination with AZD8055 monotherapy (Fig. S1B), we observed that KRAS signaling upregulation was reversed, in line with the inhibition of upstream stimulation through the RTK exerted by SHP2 co‐inhibition. Importantly, we observed that the combination was able to further downregulate MYC targets as compared with AZD8055 monotherapy. These data indicate a potent anti‐oncogenic effect of the combined mTOR and SHP2 inhibition. Of note, the synergistic effect of the two inhibitors was confirmed in terms of reduced proliferation, apoptosis induction and biochemical and transcriptional suppression of mTOR activity, also in Huh7 cells, where SHP2 reactivation could not be observed 48 h after treatment with AZD8055 (Fig. 2A). This suggests that the feedback reactivation of RTK, and consequently of SHP2, can occur with different dynamics in various HCC models but ultimately leads to the same long‐term benefit of the co‐inhibition of SHP2 and mTOR. Taken together, these data show that dual inhibition of SHP2 and mTOR produces a sustained downregulation of major oncogenic pathways with consequent induction of apoptosis.

### 3.4 Combination of mTOR and SHP2 inhibitors is effective in multiple HCC models *in vivo*

**Fig. 4:**
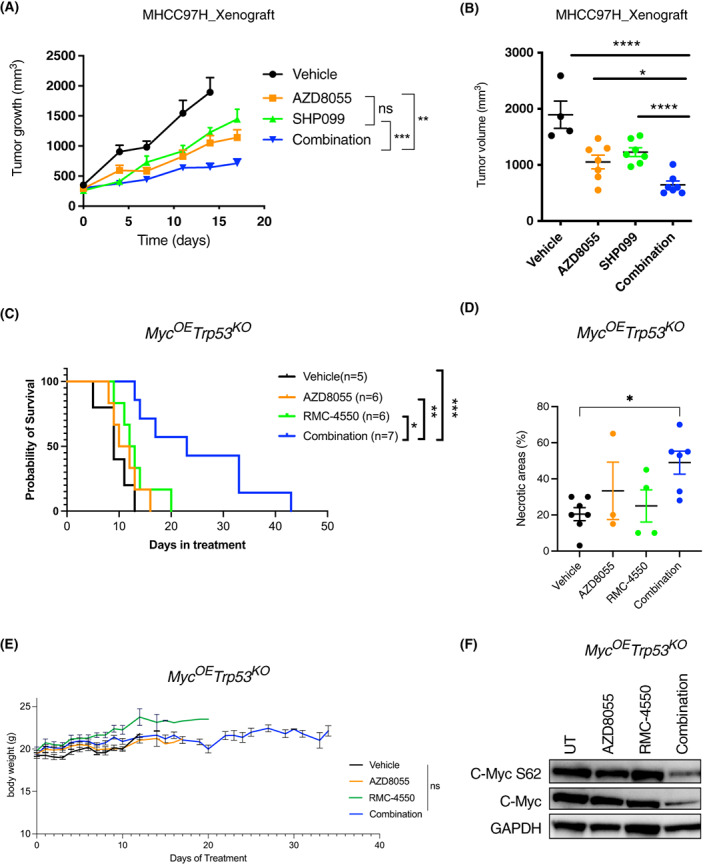
Combination of mTOR and SHP2 inhibitors is effective in several HCC models *in vivo*. (A) MHCC97H cells were grown as tumor xenografts in BALB/c nude mice. Longitudinal progression of tumor volume in mice bearing MHCC97H cells that were treated for a maximum of 17 days,with vehicle, AZD8055 (10 mg·kg^−1^), SHP099 (37.5 mg·kg^−1^) or both drugs combined. Graph shows mean ± SEM. Statistical analyses using two‐way ANOVA mixed effects test. **P < 0.01; ***P < 0.001 (vehicle *n* = 6, AZD8055 *n* = 7, SHP099 *n* = 7, combination *n* = 7). (B) MHCC97H xenograft depicting tumor volume (mm^3^) at endpoint for vehicle group (14 days). Graph shows mean ± SEM, analyzed with two‐sided unpaired Student *t*‐test. *P < 0.05; ****P < 0.0001 (vehicle *n* = 4, AZD8055 *n* = 7, SHP099 *n* = 7, combination *n* = 7). (C) Survival curve generated from *Myc* ^OE^;*Trp53* ^KO^ tumor‐bearing mice treated with vehicle (9 days), AZD8055 (10 mg·kg^−1^) (11 days), RMC‐4550 (30 mg·kg^−1^) (12.5 days) or combination (23 days). Statistical analyses using log‐rank (Mantel–Cox) test. *P < 0.05; **P < 0.01; ***P < 0.001 (vehicle *n* = 5, AZD8055 *n* = 6, RMC‐4550 *n* = 6, combination *n* = 7). (D) Quantitative blind scoring analyses of percentage of necrotic areas at endpoint in the different groups from experiment in Fig. 4C. Statistical analyses using a one‐way ANOVA test with multiple comparisons. *P < 0.05. Error bars indicate standard deviation (SD) (vehicle *n* = 7, AZD8055 *n* = 3, RMC‐4550 *n* = 4, combination *n* = 6). (E) Graph from the previous model measuring bodyweight over the days of treatment as marker for tolerability to the different treatment groups depicted. Statistical analyses using two‐way ANOVA mixed effects test. Error bars indicate standard deviation (SD) (vehicle *n* = 4, AZD8055 *n* = 6, RMC‐4550 *n* = 6, combination *n* = 7). (F) Western blot analyses from mice cells *Myc* ^OE^;*Trp53* ^KO^ untreated (UT) or treated for 72 h with AZD8055 (50 nm), RMC‐4550 (2.5 μm) or a combination of both (*n* = 2).

Both mTOR and SHP2 inhibitors have been tested in preclinical studies [27, 45, 46, 47, 48, 50], either alone or in combination with other drugs. Whereas mTOR inhibitors have been studied for a long time, and some have already been approved for the treatment of specific malignancies [51, 52, 53, 54], SHP2 inhibitors are more recent and are still being tested in earlier phases of clinical studies [55, 56, 57]. Since our *in vitro* data showed promising synergy, we tested the efficacy of the combination *in vivo*. We injected human MHCC97H cells subcutaneously in immunocompromised mice, and compared tumor growth in mice receiving vehicle, AZD8055, the SHP2 inhibitor SHP099 (whose structure is similar to compound #57, but is suitable for *in vivo* studies) or a combination of both inhibitors. The combination of mTOR and SHP2 inhibitors induced a more potent delay in tumor growth as judged by both tumor growth (Fig. 4A) and tumor volume (Fig. 4B). Next, we used a somatic, immunocompetent HCC model based on *Myc* overexpression combined with *Trp53* deletion specifically in hepatocytes [25, 58]. Tumor cells derived from this *Myc* ^OE^;*Trp53* ^KO^ mouse model were tested *in vitro* to confirm synergistic effect of combining RMC‐4550 and AZD8055 (Fig. S5A), increased SHP2 phosphorylation (Y542) upon mTOR inhibition (Fig. S5B) as well as apoptosis induction (Fig. S5C,D). *In vivo*, this aggressive HCC model quickly developed multinodular HCC tumors with dedifferentiated characteristics, as previously reported [25, 58]. We then compared tumor growth in the presence of vehicle, AZD8055, RMC‐4550 or the combination of the last two, in mice that developed HCC after hydrodynamic tail vein injection of the *Myc* ^OE^;*Trp53* ^KO^ vectors, as described previously [26]. Strikingly, the combination of both drugs significantly improved the survival of the mice (Fig. 4C). In detail, we observed that the combination group showed substantially improved median survival (23 days) when compared with RMC‐4550 (12.5 days), AZD8055 (11 days) or vehicle (9 days). At the end of the experiment, large necrotic areas were observed in residual tumors from mice treated with the combination therapy (Fig. 4D and Fig. S5F). Importantly, the drugs were well tolerated, as judged by bodyweight measurements (Fig. 4E ).

It has been proposed previously that mTOR activity is necessary for MYC‐driven hepatocarcinogenesis [59]. Thus, we checked whether the strong suppression of mTOR activity achieved by the combined inhibition of mTOR and SHP2, could impair the proliferation of *Myc* ^OE^;*Trp53* ^KO^ tumor cells by downregulating MYC itself [60]. Indeed, we observed that, *in vitro*, those cells show decreased MYC levels when treated with the combination for 72 h (Fig. 4F). Furthermore, western blot analyses of the tumors collected at endpoint from the *in vivo* experiment, revealed that two of three mice treated with the combination had decreased MYC expression, as compared with mice receiving vehicle or monotherapies (Fig. S5E). Importantly, those two mice had better survival and lower tumor volume as compared with the mouse with stable MYC levels, suggesting that MYC downregulation could be a potential marker for anti‐tumor response to the combined therapy. These observations are in line with the downregulation of MYC targets observed in the gene expression analysis of multiple human HCC cell lines (Fig. S1). There we observed that mTOR inhibitor monotherapy was already able to downregulate MYC targets, whereas KRAS signaling was upregulated (likely as a consequence of RTK overexpression feedback) (Fig. S1A). On the other hand, when inhibiting both mTOR and SHP2, MYC targets are further downregulated (Fig. S1B), which could be the result of improved mTOR suppression, but also of inhibition of the parallel RAS‐MAPK pathway. These data indicate that effectively blocking RAS‐MAPK and mTOR‐PI3K‐AKT signaling by combining SHP2 and mTOR inhibitors, promotes MYC degradation and downregulation, exerting a potent anti‐tumor effect.

It has been previously reported that SHP2 inhibition can boost the anti‐tumor immune response by impairing PD1 signaling [61]. We therefore investigated whether the combined treatment with AZD8055 and RMC4550 improved survival of *Myc* ^OE^;*Trp53* ^KO^ by increasing the anti‐tumor immune response. In‐depth flow cytometry analyses of myeloid and lymphoid cell populations showed that their abundance was not significantly altered in either of the monotherapy treatment regimens or in the combination therapy, as compared with vehicle (Fig. S6A,B). Moreover, the phenotype of conventional CD4^+^ and CD8^+^ T cells was not altered following SHP2 inhibition, and no visible immune boosting effects were observed with RMC‐4550 monotherapy. The activation of these adaptive immune cells had a mild effect on mTOR inhibition, but this effect was not altered by the addition of the SHP2 inhibitor (Fig. S6C,D). These results suggest that the survival benefit observed with the combination treatment is a tumor cell‐intrinsic anti‐cancer response and that the immune system is only marginally involved.

Altogether, these data show the feasibility and efficacy of the double inhibition of mTOR and SHP2 in HCC models, and supports further clinical exploration of the proposed combination for the treatment of hepatocellular carcinoma.

## 4 Conclusions

In the last decade, several new therapies have been implemented for the treatment of HCC [62], although those patients who are not eligible for surgery still have a poor prognosis [63]. In this report, we observe the activation of multiple RTK following treatment with AZD8055 in HCC, leading to SHP2 activation. We have further shown that SHP2 depletion or inhibition sensitizes HCC tumors to mTOR inhibition. We tested the combination of several SHP2 inhibitors with AZD8055, both *in vitro* and *in vivo*. The combination proved to have a strong synergistic anti‐cancer effect by triggering apoptosis, in contrast to the weak cytostatic effect of the monotherapies. Importantly, the combined treatment was well tolerated and significantly improved the survival in mice with aggressive HCC. Further clinical studies are needed to test the tolerability and efficacy of this combination as a new treatment strategy for patients suffering from HCC.

## Conflict of interest

The authors declare no competing interests.

## Author contributions

AMS, CW, SM and RB designed the study. *In vitro* cell culture and colony formation assays were performed by AMS and AdC. qRT‐PCR were done by AdC and ABo. Synergy matrix experiments were performed by AMS and SJIK. BLISS analyses were done and discussed with CL and RLB. RNA sequencing analyses were done and discussed with CL and RLB. SHP2 KO were generated by AMS. Apoptosis assay and immunoblotting were done by AMS, AdC and SJIK. *In vitro* data was analyzed by AMS, AdC and SJIK. AMS, CFAR and LA designed the immunocompetent somatic mouse model experiment. CFAR and MHPG conducted the immunocompetent somatic mouse model experiment. CFAR analyzed the immunocompetent somatic mouse model. ABu prepared the drugs and vehicle for the orthotopic mouse model experiment. AMS designed the xenograft experiment. CW and HW performed and analyzed the xenograft mouse model experiment. JYS did the analyses for necrotic areas. AMS, SM and RB selected the data shown in this manuscript. AMS generated the figures with the help of SM and RB. AMS wrote the paper and SM and RB edited it. Supervision and discussions were provided by RLB, CW, OvT, RB, LA and SM.

### Peer review

The peer review history for this article is available at https://publons.com/publon/10.1002/1878‐0261.13377.

## Supporting information


**Fig. S1.** GSEA analyses of 50 hallmarks gene sets for cancer. GSEA analyses from RNA sequencing data. The mean of the NES scores of the three lines is shown and the table is sorted on this mean column in decreasing order. (A) Huh7, Hep3B and MHCC97H comparing cells treated with AZD8055 to untreated cells. (B) Huh7, Hep3B and MHCC97H comparing cells treated with combination of AZD8055 plus compound #57 to AZD8055 monotherapy. *P*‐values represented as: * *P* = 0.01 < *P* ≤ 0.05; ** *P* = 0.001 < *P* ≤ 0.01; *** *P* ≤ 0.001.

**Fig. S2.** MHCC97H *PTPN11* KO cells have diminished mTOR reactivation and are more sensitive to AZD8055. (A) MHCC97H WT and *PTPN11* KO cells were treated with 50 nm AZD8055. Samples were collected at indicated time points to blot for the indicated antibodies. Untreated was collected at 24 h. (B) MHCC97H WT and *PTPN11* knockout cells were grown in the absence or presence of AZD8055. Phase‐contrast images were taken every 4 h and cell proliferation was determined based on cell confluence. Graphs show mean ± standard deviation. Statistical analyses using unpaired *t‐*test. **P < 0.01; ****P < 0.0001. (C,D) Densitometries measuring the intensity of phosphorylation over time in: 4EBP1 S65, 4EBP1 T37/46, S6RP S235/236 and S6RP S240/244 in Hep3B WT (C) or MHCC97H (D) vs PTPN11 KO cells. Dashes line is above the 72‐h timepoint of the parental cells.

**Fig. S3.** Combination of AZD8055 (mTORi) with both RMC‐4550 and compound #57, (SHP2 inhibitors) is synergistic in HCC models *in vitro*. BLISS score analyses highlight with a green background the combinatorial doses for which we find significant values of synergy. The delta value, percentage and *P*‐value are depicted in the figures. (A) Matrix for synergy with increasing doses of AZD8055 and RMC‐4550 inhibitor in Hep3B and MHCC97H cell lines. (B) Matrix for synergy with increasing doses of AZD8055 and compound #57 inhibitor in Hep3B and MHCC97H cell lines.

**Fig. S4.** AZD8055 and SHP2 co‐inhibition triggers apoptosis in several HCC models. (A) Representative images of the apoptosis assay shown in Fig. 3C. All pictures were taken using x10 lens. Scale bar: 80 μm. (B) Quantitative measurement of Caspase 3/7 reagent being detected over time in four different HCC cell lines at the indicated doses. Combination = AZD8055 (100 nm) plus Compound #57 (5 μm). (C) Quantitative measurement of Caspase 3/7 reagent being detected at 48 h in two different HCC cell lines at the indicated doses. Combination = AZD8055 (100 nm) plus RMC‐4550 (5 μm). Statistical analyses using a one‐way ANOVA test, *P < 0.05; ***P < 0.001; ****P < 0.0001.

**Fig. S5.** *Myc* ^OE^;*Trp53* ^KO^ mouse cells behave similarly to human HCC models. (A) Matrix for synergy with increasing doses of an mTOR and a SHP2 inhibitor. BLISS score analyses highlight with a green background the combinatorial doses for which we find significant values of synergy. The delta value, percentage and *P*‐value are depicted in the figures. (B) *Myc* ^OE^;*Trp53* ^KO^ mouse cells were treated with AZD8055 (50 nm), and cell lysates including those from untreated (UT) cells were collected at 48 h to blot for the indicated antibodies. (C) Representative images of the apoptosis assay at 72 h treated with the indicated doses. Quantitative measurement of Caspase 3/7 reagent being detected over time in four different HCC cell lines at the indicated doses. All pictures were taken using x10 lens. Scale bar: 300 μm. (D) Quantitative apoptosis assay measuring Caspase 3/7 activity at 72 h in the different treatment groups (*n* = 1). Combinatio*n* = AZD8055 (100 nm) plus Compound #57 (5 μm). (E) Western blot analyses from lysates extracted from tumors of the experiment shown in Fig. 4C (endpoint). Blotting for c‐MYC and c‐MYC S62 with GAPDH as loading control. Comparison between three different mice per arm (*n* = 12). Table indicating the tumor volume, days of treatment and intensity of MYC and p‐MYC levels in the three different mice treated with the combination that are tested in the same western blot. (F) Representative picture of hematoxylin & eosin (H&E) staining for the different groups from experiment (C). All pictures were taken using ×5 lens. Scale bar: 200 μm.

**Fig. S6.** Flow cytometry quantification of lymphoid and myeloid populations and activity markers within the liver of mice harboring somatic multinodular HCC tumors. (A) Myeloid populations were quantified by FACS. For the myeloid population, the sample size was as follows at the endpoint: *n* = 5 vehicle‐treated mice, *n* = 4 AZD8055‐treated mice, *n* = 5 RMC‐4550‐treated mice and *n* = 3 combination‐treated mice. Data are mean SEM statistical test: Unpaired Student's *t*‐test. Monocytes (CD45^+^CD11b^+^Ly6G^−^Ly6C^+^), TAM = tumor‐associated macrophages (CD45^+^CD11b^+^Ly6C^−^Ly6G^−^F4/80^+^), BMDM = bone marrow derived macrophages (CD45^+^CD11b^hi^Ly6C^−^Ly6G^−^F4/80^int^), KC = Kupffer cells (CD45^+^CD11b^int^Ly6C^−^Ly6G^−^F4/80^hi^), neutrophils (CD45^+^CD11b^+^Ly6C^int^Ly6G^+^), DC = dendritic cells (CD45^+^F4/80^−^CD11c^+^MHCII^+^). (B) Lymphoid populations were quantified by FACS. For the lymphoid population, the sample size was as follows at the endpoint: *n* = 4 vehicle‐treated mice, *n* = 3 AZD8055‐treated mice, *n* = 5 RMC‐4550‐treated mice and *n* = 3 combination‐treated mice. Data are mean SEM, statistical test: Unpaired Student's *t*‐test. NK = Natural killer cells (CD45^+^CD3^−^CD19^−^NK1.1^+^), NKT = Natural killer T‐lymphocyte cells (CD45^+^CD3^+^CD19^−^NK1.1^+^), B cells (CD45^+^CD3^−^CD19^+^NK1.1^−^), CD4T = CD4^+^ T‐cells (CD45^+^CD3^+^NK1.1^−^CD19^−^CD4^+^), Conv CD4T = conventional CD4^+^ T‐cells (CD45^+^CD3^+^NK1.1^−^CD19^−^CD4^+^FOXP3^‐^), Tregs = regulatory T‐cells (CD45^+^CD3^+^NK1.1^−^CD19^−^CD4^+^FOXP3^+^) and CD8+ T cells (CD45^+^CD3^+^NK1.1^−^CD19^−^CD8^+^). (C,D) Bar plots depicting the percentage of cells expressing the phenotypic and activation markers in CD8T cells and conventional CD4T cells. Graphs show mean ± SEM. Statistical significance was determined by unpaired Student's *t*‐test. *P < 0.05; **P < 0.01; ****P < 0.0001.

**Table S1.** Panel of antibodies used for flow cytometry. Orange, myeloid panel: Depicts markers for cell surface, intracellular and a control for live/dead cells followed by the clone for the antibody, the product reference and the dilution used for each antibody. Green, lymphoid panel: Depicts markers for cell surface, intracellular and a control for live/dead cells followed by the clone for the antibody, the product reference and the dilution used for each antibody.

## Data Availability

Supplementary figures are available in this manuscript. RNAseq data were uploaded to GEO under the reference Series GSE217810 and can be accessed via GEO: GSE217810. This paper does not report original code. Any additional information required to reanalyze the data reported in this work paper is available from the lead contact upon request.
