# Supplementary figures and images for: Rational combination of SHP2 and mTOR inhibition for the treatment of hepatocellular carcinoma
Source: Mol Oncol. 2023 Feb 9;17(6):964–80. doi: 10.1002/1878-0261.13377 (PMC10257427; doi:10.1002/1878-0261.13377)

Supplementary Figure 1

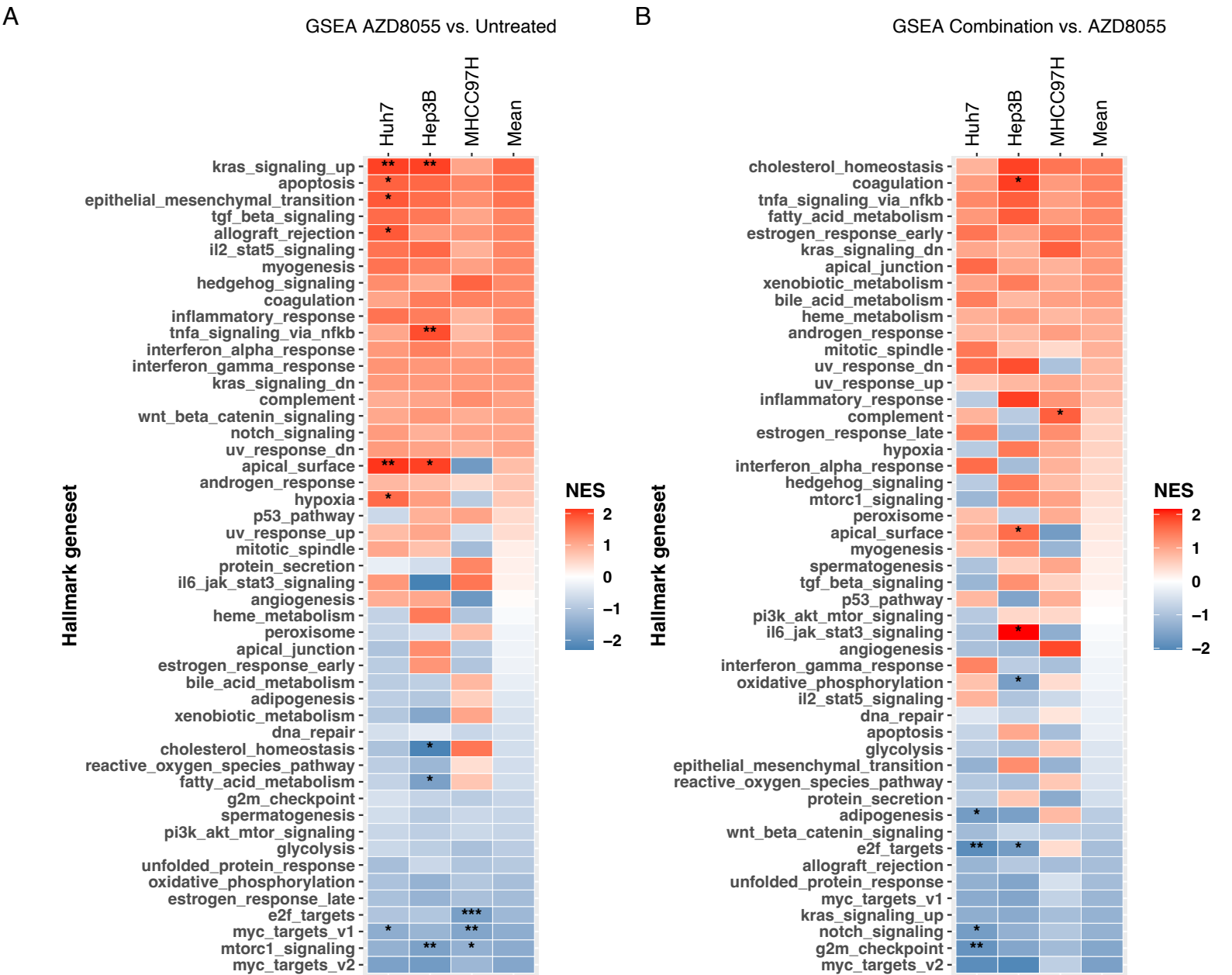

Supplement: Supplementary file 1 — Fig. S1. GSEA analyses of 50 hallmarks gene sets for cancer. GSEA analyses from RNA sequencing data. The mean of the NES scores of the three lines is shown and the table is sorted on this mean column in decreasing order. (A) Huh7, Hep3B and MHCC97H comparing cells treated with AZD8055 to untreated cells. (B) Huh7, Hep3B and MHCC97H comparing cells treated with combination of AZD8055 plus compound #57 to AZD8055 monotherapy. P‐values represented as: * P = 0.01 < P ≤ 0.05; ** P = 0.001 < P ≤ 0.01; *** P ≤ 0.001. [file MOL2-17-964-s006.pdf]

Supplementary Figure 2

A

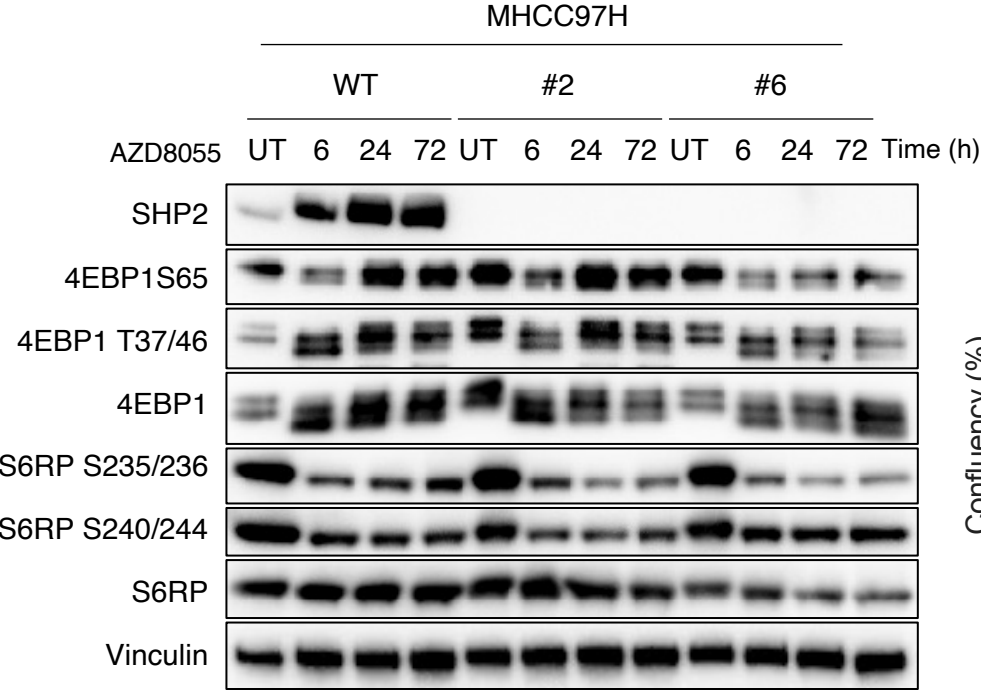

B

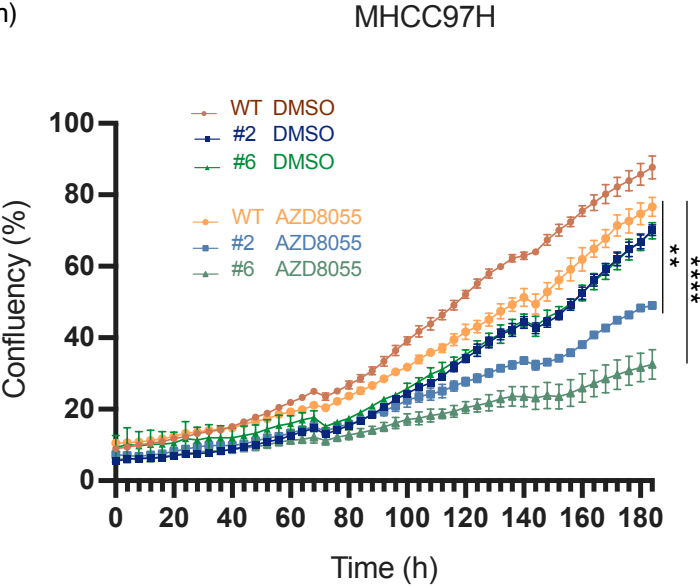

C

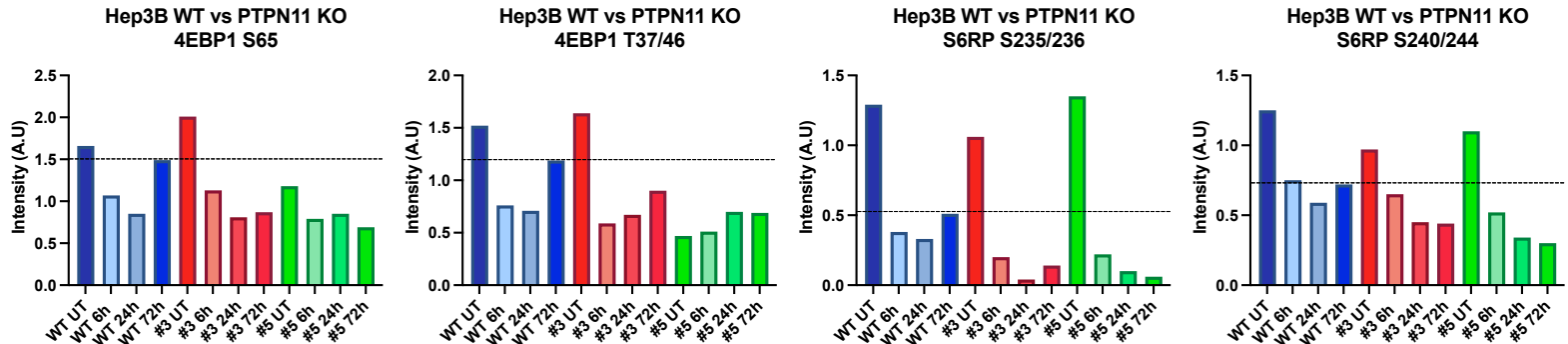

D

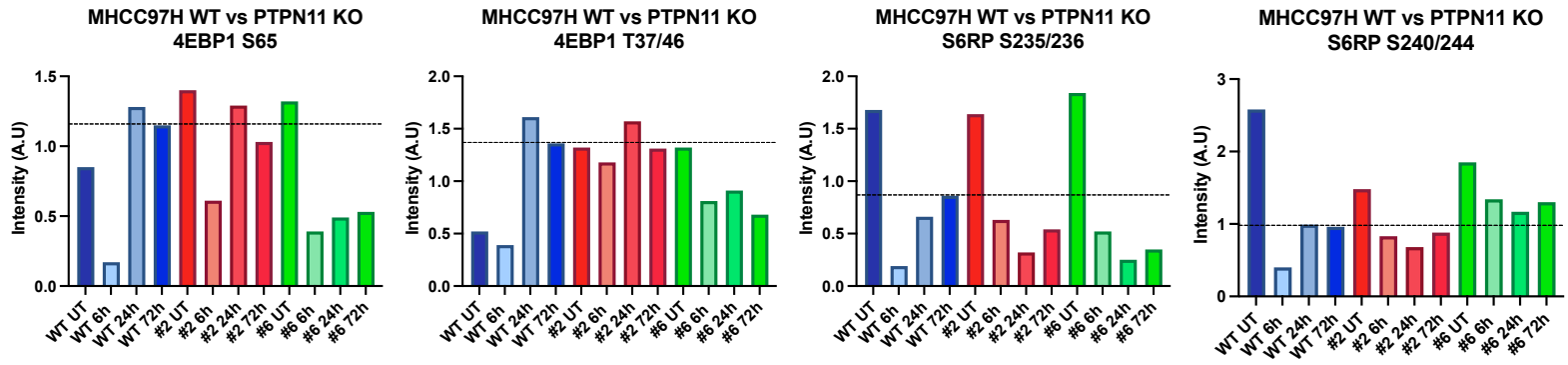

Supplement: Supplementary file 2 — Fig. S2. MHCC97H PTPN11 KO cells have diminished mTOR reactivation and are more sensitive to AZD8055. (A) MHCC97H WT and PTPN11 KO cells were treated with 50 nm AZD8055. Samples were collected at indicated time points to blot for the indicated antibodies. Untreated was collected at 24 h. (B) MHCC97H WT and PTPN11 knockout cells were grown in the absence or presence of AZD8055. Phase‐contrast images were taken every 4 h and cell proliferation was determined based on cell confluence. Graphs show mean ± standard deviation. Statistical analyses using unpaired t‐test. **P < 0.01; ****P < 0.0001. (C,D) Densitometries measuring the intensity of phosphorylation over time in: 4EBP1 S65, 4EBP1 T37/46, S6RP S235/236 and S6RP S240/244 in Hep3B WT (C) or MHCC97H (D) vs PTPN11 KO cells. Dashes line is above the 72‐h timepoint of the parental cells. [file MOL2-17-964-s007.pdf]

Supplementary Figure 3

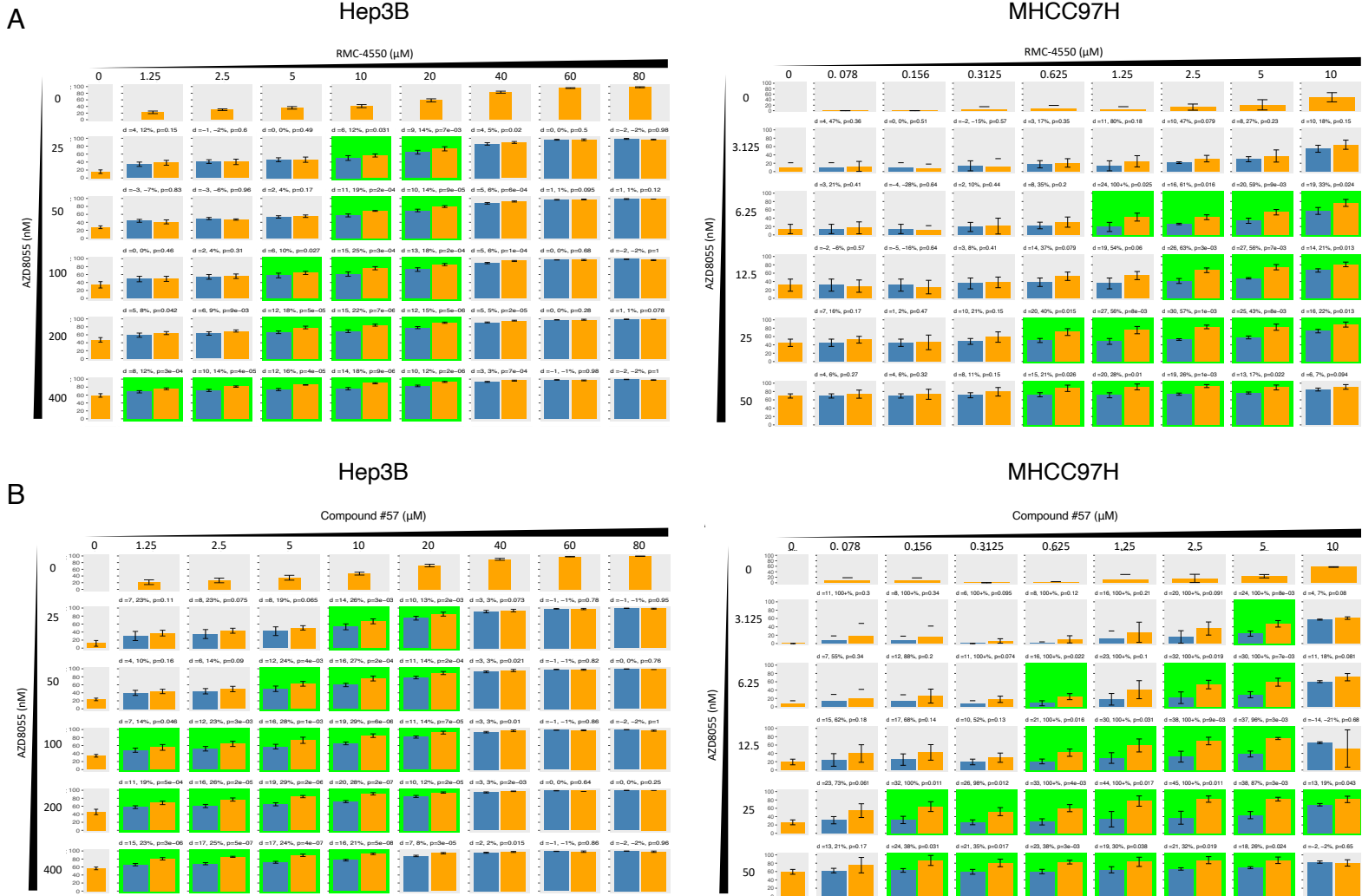

Supplement: Supplementary file 3 — Fig. S3. Combination of AZD8055 (mTORi) with both RMC‐4550 and compound #57, (SHP2 inhibitors) is synergistic in HCC models in vitro. BLISS score analyses highlight with a green background the combinatorial doses for which we find significant values of synergy. The delta value, percentage and P‐value are depicted in the figures. (A) Matrix for synergy with increasing doses of AZD8055 and RMC‐4550 inhibitor in Hep3B and MHCC97H cell lines. (B) Matrix for synergy with increasing doses of AZD8055 and compound #57 inhibitor in Hep3B and MHCC97H cell lines. [file MOL2-17-964-s003.pdf]

Supplementary Figure 4

caspase-3/7 apoptosis assay (72h)

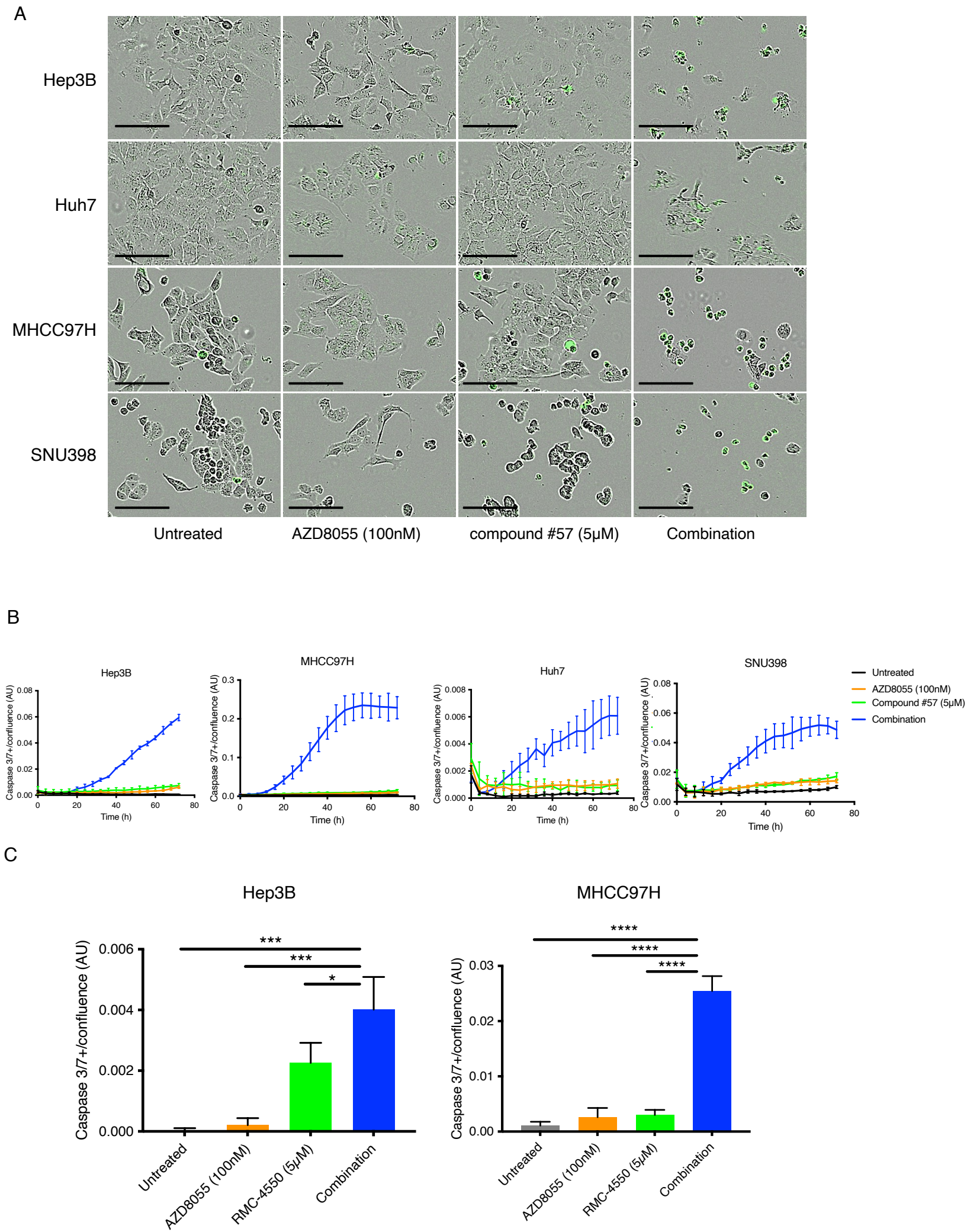

Supplement: Supplementary file 4 — Fig. S4. AZD8055 and SHP2 co‐inhibition triggers apoptosis in several HCC models. (A) Representative images of the apoptosis assay shown in Fig. 3C. All pictures were taken using x10 lens. Scale bar: 80 μm. (B) Quantitative measurement of Caspase 3/7 reagent being detected over time in four different HCC cell lines at the indicated doses. Combination = AZD8055 (100 nm) plus Compound #57 (5 μm). (C) Quantitative measurement of Caspase 3/7 reagent being detected at 48 h in two different HCC cell lines at the indicated doses. Combination = AZD8055 (100 nm) plus RMC‐4550 (5 μm). Statistical analyses using a one‐way ANOVA test, *P < 0.05; ***P < 0.001; ****P < 0.0001. [file MOL2-17-964-s005.pdf]

Supplementary Figure 5

A

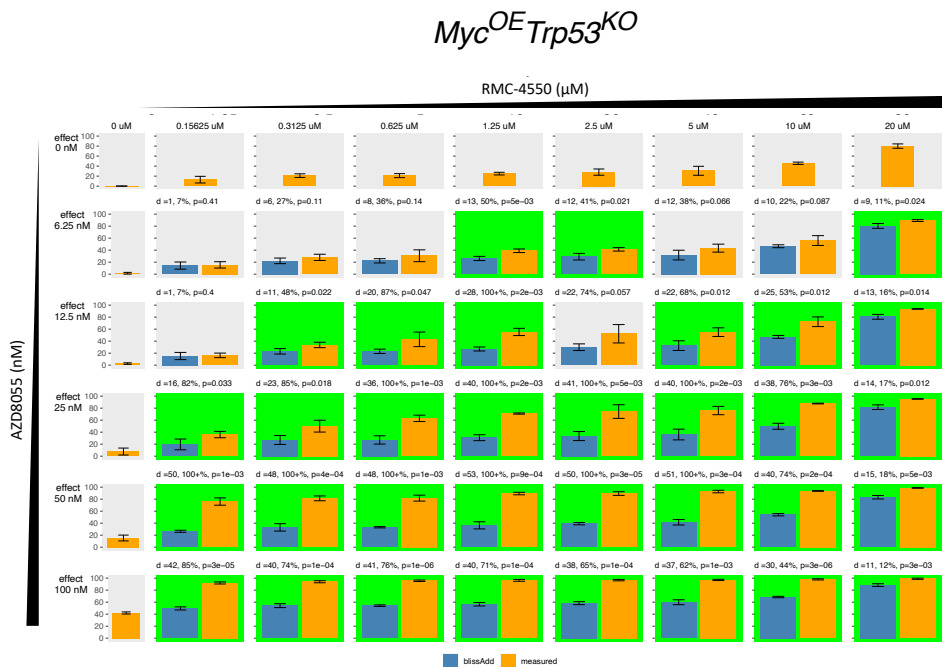

B

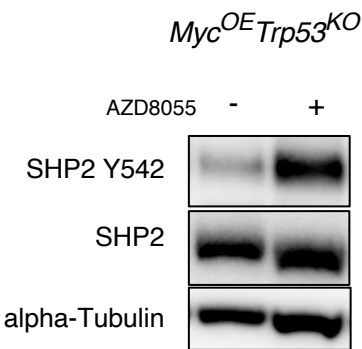

C

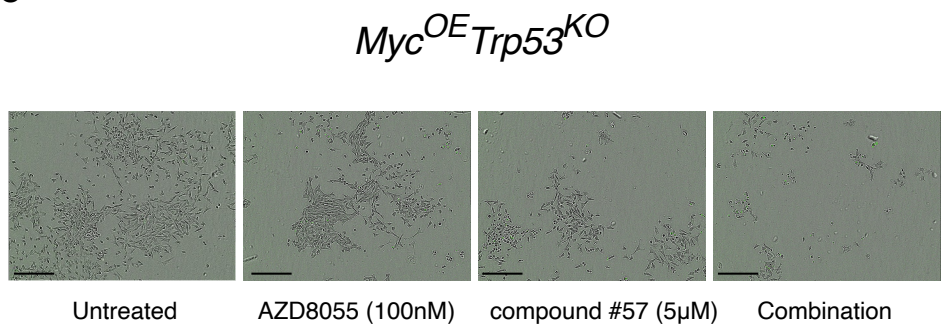

D

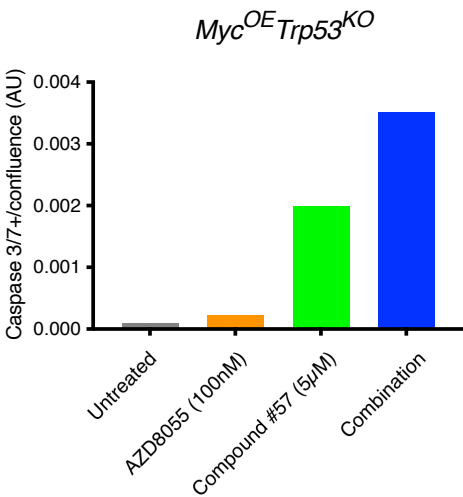

E

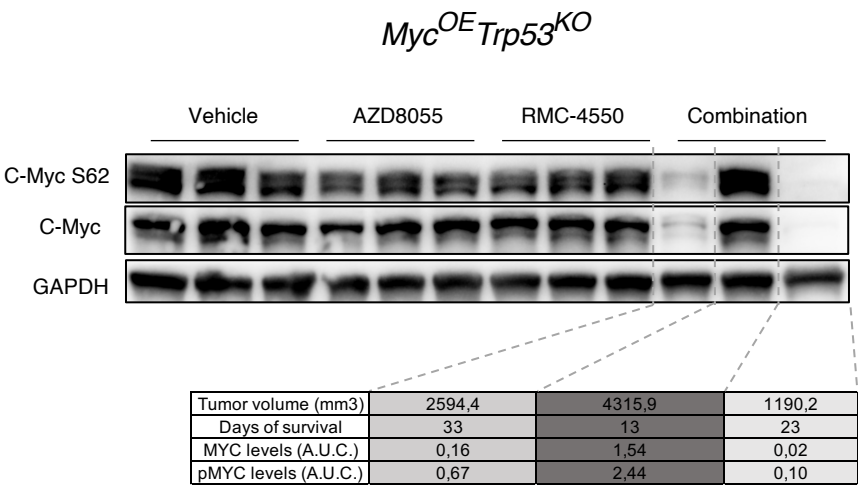

F

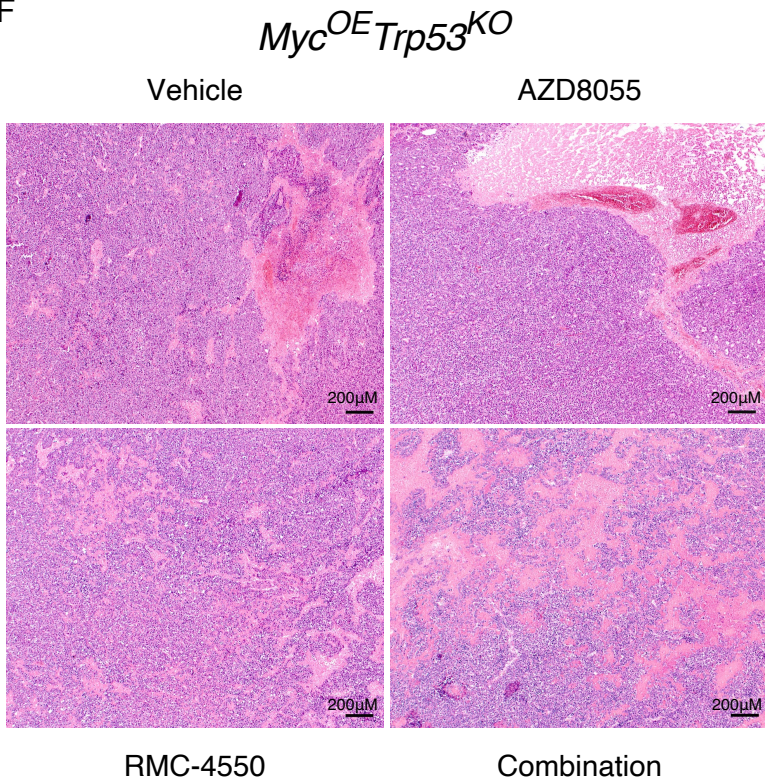

Supplement: Supplementary file 5 — Fig. S5. Myc OE;Trp53 KO mouse cells behave similarly to human HCC models. (A) Matrix for synergy with increasing doses of an mTOR and a SHP2 inhibitor. BLISS score analyses highlight with a green background the combinatorial doses for which we find significant values of synergy. The delta value, percentage and P‐value are depicted in the figures. (B) Myc OE;Trp53 KO mouse cells were treated with AZD8055 (50 nm), and cell lysates including those from untreated (UT) cells were collected at 48 h to blot for the indicated antibodies. (C) Representative images of the apoptosis assay at 72 h treated with the indicated doses. Quantitative measurement of Caspase 3/7 reagent being detected over time in four different HCC cell lines at the indicated doses. All pictures were taken using x10 lens. Scale bar: 300 μm. (D) Quantitative apoptosis assay measuring Caspase 3/7 activity at 72 h in the different treatment groups (n = 1). Combination = AZD8055 (100 nm) plus Compound #57 (5 μm). (E) Western blot analyses from lysates extracted from tumors of the experiment shown in Fig. 4C (endpoint). Blotting for c‐MYC and c‐MYC S62 with GAPDH as loading control. Comparison between three different mice per arm (n = 12). Table indicating the tumor volume, days of treatment and intensity of MYC and p‐MYC levels in the three different mice treated with the combination that are tested in the same western blot. (F) Representative picture of hematoxylin & eosin (H&E) staining for the different groups from experiment (C). All pictures were taken using ×5 lens. Scale bar: 200 μm. [file MOL2-17-964-s002.pdf]

Supplementary Figure 6

A

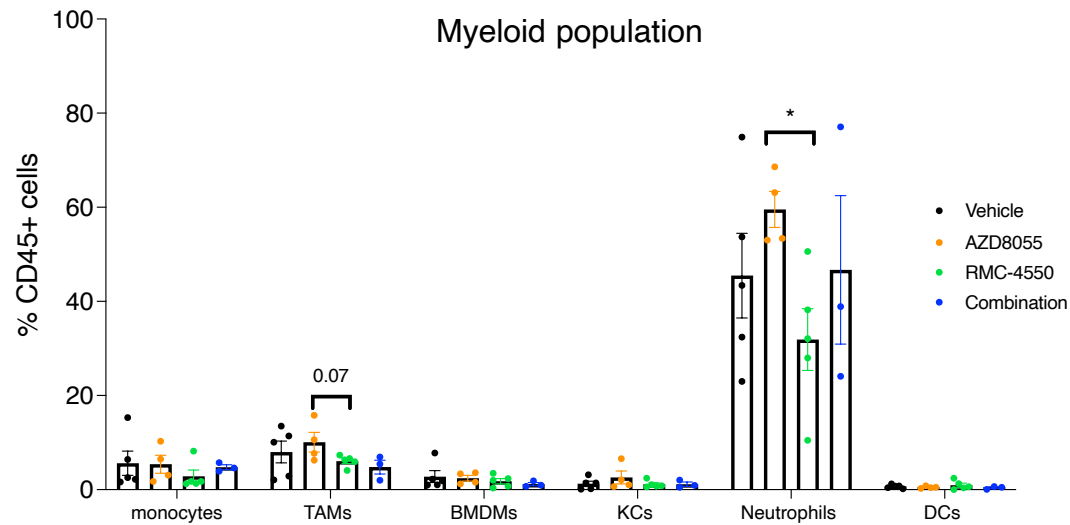

B

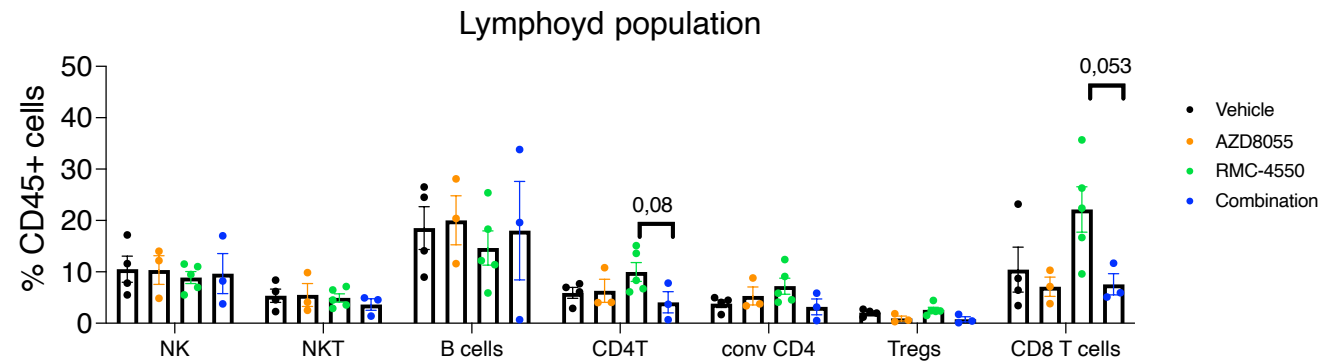

C

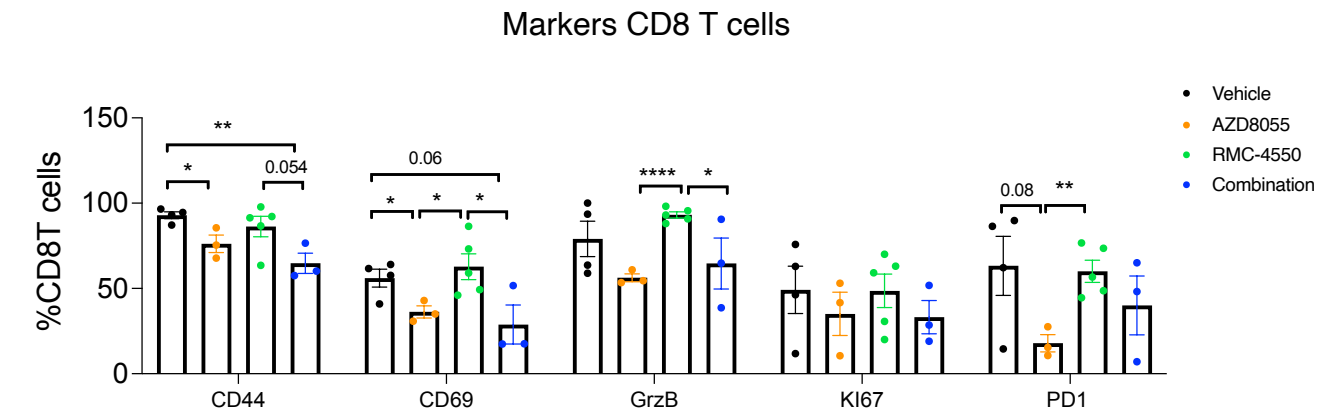

D

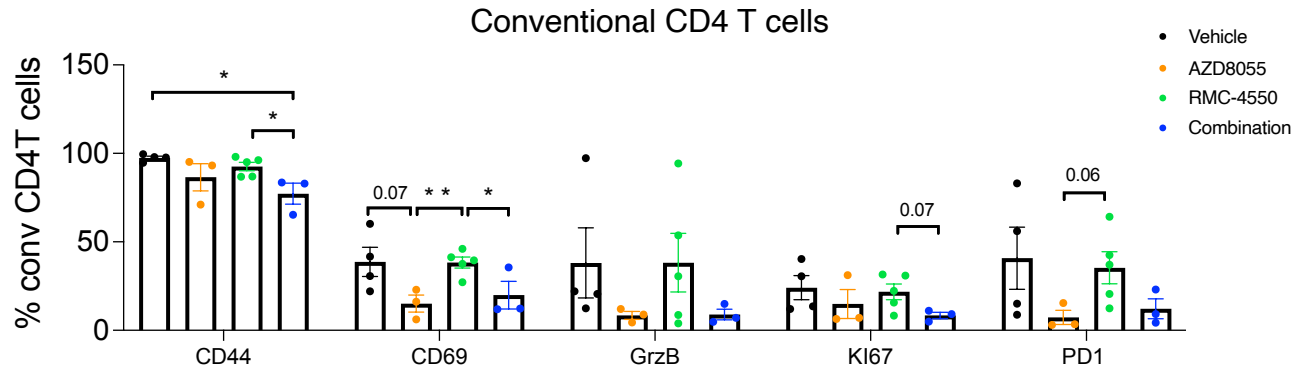

Supplement: Supplementary file 6 — Fig. S6. Flow cytometry quantification of lymphoid and myeloid populations and activity markers within the liver of mice harboring somatic multinodular HCC tumors. (A) Myeloid populations were quantified by FACS. For the myeloid population, the sample size was as follows at the endpoint: n = 5 vehicle‐treated mice, n = 4 AZD8055‐treated mice, n = 5 RMC‐4550‐treated mice and n = 3 combination‐treated mice. Data are mean SEM statistical test: Unpaired Student's t‐test. Monocytes (CD45+CD11b+Ly6G−Ly6C+), TAM = tumor‐associated macrophages (CD45+CD11b+Ly6C−Ly6G−F4/80+), BMDM = bone marrow derived macrophages (CD45+CD11bhiLy6C−Ly6G−F4/80int), KC = Kupffer cells (CD45+CD11bintLy6C−Ly6G−F4/80hi), neutrophils (CD45+CD11b+Ly6CintLy6G+), DC = dendritic cells (CD45+F4/80−CD11c+MHCII+). (B) Lymphoid populations were quantified by FACS. For the lymphoid population, the sample size was as follows at the endpoint: n = 4 vehicle‐treated mice, n = 3 AZD8055‐treated mice, n = 5 RMC‐4550‐treated mice and n = 3 combination‐treated mice. Data are mean SEM, statistical test: Unpaired Student's t‐test. NK = Natural killer cells (CD45+CD3−CD19−NK1.1+), NKT = Natural killer T‐lymphocyte cells (CD45+CD3+CD19−NK1.1+), B cells (CD45+CD3−CD19+NK1.1−), CD4T = CD4+ T‐cells (CD45+CD3+NK1.1−CD19−CD4+), Conv CD4T = conventional CD4+ T‐cells (CD45+CD3+NK1.1−CD19−CD4+FOXP3‐), Tregs = regulatory T‐cells (CD45+CD3+NK1.1−CD19−CD4+FOXP3+) and CD8+ T cells (CD45+CD3+NK1.1−CD19−CD8+). (C,D) Bar plots depicting the percentage of cells expressing the phenotypic and activation markers in CD8T cells and conventional CD4T cells. Graphs show mean ± SEM. Statistical significance was determined by unpaired Student's t‐test. *P < 0.05; **P < 0.01; ****P < 0.0001. [file MOL2-17-964-s001.pdf]
